# Supplementary material for: Comprehensive Assessment of Force-Field Performance in Molecular Dynamics Simulations of DNA/RNA Hybrid Duplexes
Source: J Chem Theory Comput. 2024 Jul 16;20(15):6917–29. doi: 10.1021/acs.jctc.4c00601 (PMC11325551; doi:10.1021/acs.jctc.4c00601)
Supplement: Supplementary file 1 — ct4c00601_si_001.pdf [file ct4c00601_si_001.pdf]

SUPPORTING INFORMATION

Comprehensive Assessment of Force-Field Performance in Molecular Dynamics Simulations of DNA/RNA Hybrid Duplexes

Barbora Knappeová[1,&], Vojtěch Mlýnský[1], Martin Pykal[2], Jiří Šponer[1], Pavel Banáš[2], Michal Otyepka[2,3], and Miroslav Krepl[1,*,&]

[1]Institute of Biophysics of the Czech Academy of Sciences, Královopolská 135, 612 00 Brno, Czech Republic

[2]Czech Advanced Technology and Research Institute, CATRIN, Palacký University, Křížkovského 511/8, Olomouc 779 00, Czech Republic

[3]IT4Innovations, VSB-Technical University of Ostrava, 17. listopadu 2172/15, 708 00 Ostrava-Poruba, Czech Republic

[&]Joint-first authors.

*Corresponding author: Miroslav Krepl
E-mail: krepl@ibp.cz

Supporting Information Text

Size of the bins for the histograms. The size of the histogram bins for the individual parameters was 5 (dihedrals) or 1 (inclination, buckle and x-displacement). The binning range was adjusted for each parameter so that all the populated values in every simulation could be binned.

Flat-well distance restraint functions applied on DNA sugars to enforce C3′-endo puckers. In one specific set of simulations (see the main text Methods and Table 1), the following restraint functions (AMBER format) were applied on each sugar ring to maintain the sugar pucker in the range of -10 to 40°. Example for the first nucleotide of the DNA strand in DD_hybrid system is shown. Note that the strength of the restraints can be freely adjusted and fine-tuned to obtain the desired C3′-endo/C2′-endo balance.

```
# 13 DC NU0:  (13 DC5 C4')-(13 DC5 O4')-(13 DC5 C1')-(13 DC5 C2') -15.0  15.0
 &rst    iat =   391,   393,   394,   410,
             r1 = -16.0, r2 = -15.0, r3 =  15.0, r4 =  16.0,
             rk2 =    2000.0, rk3 =   2000.0,                              /

# 13 DC NU1:  (13 DC5 O4')-(13 DC5 C1')-(13 DC5 C2')-(13 DC5 C3') -36.5  -6.5
 &rst    iat =   393,   394,   410,   408,
             r1 = -37.5, r2 = -36.5, r3 =  -6.5, r4 =  -5.5,
           /

# 13 DC NU2:  (13 DC5 C1')-(13 DC5 C2')-(13 DC5 C3')-(13 DC5 C4')  19.8  49.8
```

```
 &rst    iat =   394,   410,   408,   391,
         r1 =  18.8, r2 =  19.8, r3 =  49.8, r4 =  50.8,
        /

# 13 DC NU3:  (13 DC5 C2')-(13 DC5 C3')-(13 DC5 C4')-(13 DC5 O4') -49.8 -19.8
 &rst    iat =   410,   408,   391,   393,
         r1 = -50.8, r2 = -49.8, r3 = -19.8, r4 = -18.8,
        /

# 13 DC NU4:  (13 DC5 C3')-(13 DC5 C4')-(13 DC5 O4')-(13 DC5 C1')   6.5  36.5
 &rst    iat =   408,   391,   393,   394,
         r1 =   5.5, r2 =   6.5, r3 =  36.5, r4 =  37.5,
```

Supporting Information Tables

Table S1. Selected helical parameters observed in the hybrid duplex structures deposited in the PDB database.[a]

PDB code	method	Sequence[b]	Buckle	Propeller	Inclination	x-displacement
479D	X-ray	DNA/RNA	5.70	-11.55	14.03	-3.98
404D	X-ray	RNA/DNA	-7.96	-7.50	10.36	-4.56
3SSF	X-ray	RNA/DNA	-5.10	-16.65	16.36	-3.30
1FIX	X-ray	RNA/DNA	-6.20	-13.67	15.19	-4.01
1NXR	NMR	DNA/RNA	5.74	-12.82	8.13	-4.25
8DRH	NMR	DNA/RNA	6.42	-15.79	16.19	-3.54
1HG9	NMR	DNA/RNA	2.53	-17.16	17.69	-3.55
124D	NMR	DNA/RNA	7.75	-12.43	11.54	-3.71
1EFS	NMR	DNA/RNA	6.32	-6.83	6.48	-3.47

[a]For solution NMR, the average values of the entire ensemble are given.
[b]Indicates whether DNA or RNA strand is positioned first in the PDB file. It affects the sign of the buckle value.

Table S2. Additional helical parameters observed in the MD simulations.

Duplex	force field DNA / RNA	Minor groove width AT / GC	Slide	Tilt	Helical rise	Tip	Helical twist
Non-polarizable force fields							
DD_DNA	OL21 / -	10.35/11.57	0.02	0.00	3.27	0.00	35.93
DD_DNA	OL15 / -	10.45/11.75	0.01	-0.01	3.27	0.01	36.10
DD_DNA	bsc1 / -	10.46/11.85	-0.23	0.01	3.25	-0.02	35.88
DD_DNA	Tumuc1 / -	9.59/10.99	0.05	0.00	3.29	-0.01	36.78
DD_DNA	DES-Amber / -	11.68/12.92	-0.58	0.00	3.24	0.00	34.19
DD_DNA	CHARMM36 / -	13.28/13.78	0.20	0.04	3.26	-0.01	35.86
DD_RNA	- / OL3	17.29/17.19	-1.56	0.00	2.63	0.01	32.71
DD_RNA	- / ROC-RNA	16.75/16.87	-1.65	0.04	2.76	-0.05	32.31
DD_RNA	- / DES-Amber	16.76/16.79	-2.03	-0.01	2.72	0.02	30.01
DD_RNA	- / CHARMM36	17.37/17.31	-1.60	0.08	2.52	-0.09	32.24
DD_hybrid	OL21 / OL3	14.91/15.19	-0.96	2.25	2.93	-3.77	33.13
DD_hybrid[a]	OL21 / OL3	17.24/16.98	-1.37	-0.36	2.72	0.64	32.95
DD_hybrid[b]	OL21 / OL3	15.01/15.23	-0.97	2.26	2.96	-3.82	33.09
DD_hybrid	OL21 / OL3 (OPC water)	15.00/15.12	-1.05	2.16	2.94	-3.66	32.97
DD_hybrid	OL15 / OL3	14.94/15.19	-0.96	2.18	2.95	-3.69	33.34
DD_hybrid[c]	OL15 / OL3	14.97/14.99	-0.92	2.06	2.97	-3.51	33.53
DD_hybrid[d]	OL15 / OL3	15.29/15.36	-1.18	2.01	2.91	-3.60	32.72
DD_hybrid	bsc1 / OL3	15.04/15.18	-1.08	2.36	2.94	-4.07	33.43
DD_hybrid[b]	bsc1 / OL3	15.09/15.24	-1.10	2.42	2.96	-4.18	33.22

DD_hybrid	bsc1 / OL3 (OPC water)	14.97/15.13	-1.16	2.32	2.94	-4.03	33.01
DD_hybrid	OL21 / ROC-RNA	14.35/14.93	-0.91	2.19	3.02	-3.91	33.51
DD_hybrid	Tumuc1 / OL3	14.84/15.17	-1.18	2.63	2.96	-4.75	33.00
DD_hybrid	Tumuc1 / ROC-RNA	14.26/14.74	-1.01	2.49	3.05	-4.55	33.42
DD_hybrid	DES-Amber / DES-Amber	15.46/15.46	-1.51	2.15	2.91	-4.14	31.22
DD_hybrid	CHARMM36 / CHARMM36	16.76/16.81	-1.17	2.14	2.82	-3.56	33.87
DD_hybrid[e]	CHARMM36 / CHARMM36	16.83/16.52	-1.21	2.25	2.81	-3.78	33.73
PPT	*X-ray structure*[f]	17.73	-1.45	-1.87	2.92	2.46	33.83
PPT	OL21 / OL3	14.29	-1.06	0.81	3.12	-1.48	32.78
PPT	OL15 / OL3	14,50	-1.12	0.94	3.09	-1.66	32.49
PPT	bsc1 / OL3	14.41	-1.22	1.01	3.10	-1.79	32.40
PPT	OL21 / ROC-RNA	13.05	-0.77	0.48	3.22	-0.89	33.85
PPT	Tumuc1 / OL3	14.12	-1.30	1.32	3.26	-2.39	32.63
PPT	DES-Amber / DES-Amber	15.03	-1.36	1.17	3.02	-2.20	31.38
PPT	CHARMM36 / CHARMM36	16.86	-1.53	-0.35	2.82	0.72	31.81
**Polarizable force fields**							
DD_DNA	DRUDE / -	12.48/13.18	-0.32	-0.01	3.28	0.02	37.98
DD_DNA	AMOEBA / -	9.88/10.52	-0.20	-0.05	3.21	0.07	35.91
DD_RNA	- / DRUDE	17.27/17.32	-1.26	0.00	2.91	0.00	34.59
DD_RNA	- / AMOEBA	16.88/16.95	-2.26	0.01	2.80	-0.03	30.83
DD_hybrid	DRUDE / DRUDE	15.49/15.96	-0.98	2.94	3.13	-4.69	35.38
DD_hybrid	AMOEBA / AMOEBA	16.77/16.14	-2.08	0.69	2.83	-1.35	30.44
PPT	DRUDE / DRUDE	14.67	-1.12	1.50	3.43	-2.63	34.27
PPT	AMOEBA / AMOEBA	15.11	-1.54	0.81	3.11	-1.62	31.85

[a]The DNA nucleotide puckers were restrained to C3′-endo region (pseudorotation values of -10° to 40°) by five flat-well dihedral restraint potential functions applied on each 2-deoxyribose (see above).

[b]The Li and Merz parameters[1] for KCl ions were utilized.

[c]The initial structure of the hybrid corresponded to the B-form helix (see Methods).

[d]The χ dihedral potentials of the OL15 DNA *ff* were replaced with the RNA χ dihedral potentials from OL3 *ff*. See Ref.[2] for more details.

[e]2 kcal/mol stabilizing sHBfix potential was applied on every base pairing H-bond.

[f]Average values observed in the PPT experimental structure[3]. See Table S1 for values observed for other experimental structures of the hybrids.

Table S3. Base pair populations (in %) in selected MD simulations (see also Figure S2).[a]

Base pair	OL21/OL3[b]				CHARMM36[b]				CHARMM36 - sHbfix[c]
	DD_DNA	DD_RNA	DD_hybrid	PPT	DD_DNA	DD_RNA	DD_hybrid	PPT	DD_hybrid
**1**	100	100	100	99	100	98	81	97	100
**2**	100	100	100	100	100	99	88	97	100
**3**	100	100	100	100	100	99	100	100	100
**4**	100	100	100	100	100	99	100	100	100
**5**	100	100	100	100	100	97	100	100	100
**6**	100	100	99	100	100	91	99	100	100
**7**	100	100	99	100	99	86	91	100	100
**8**	100	100	99	100	100	92	96	100	100
**9**	100	100	99	100	100	99	98	94	100
**10**	100	100	100	100	100	99	93	81	100
**11**	100	100	100	-	77	99	91	-	100
**12**	100	100	100	-	91	98	85	-	100

[a]The individual base pairs were considered populated as long as at least one of their Watson-Crick H-bonds was present. An H-bond was considered present when the distance between donor and acceptor was below 3.5 Å.

[b]1 kcal/mol structure-specific HBfix (sHBfix) potential[4] was used to stabilize the Watson-Crick H-bonds of the terminal base pairs. This was the standard setting utilized in all simulations (see the main text.)

[c]2 kcal/mol sHBfix was used to stabilize the Watson-Crick H-bonds of every base pair.

Table S4. Median values of the backbone dihedral angles observed in MD simulations of the PPT hybrid structure using the polarizable and selected non-polarizable force fields.[a]

Dihedrals / Force fields	RNA							DNA						
	α	β	γ	δ	ε	ζ	χ	α	β	γ	δ	ε	ζ	χ
**OL21/OL3**	284	170	65	81	200	290	202	295	175	52	125	184	268	241
**CHARMM36**	286	173	63	77	204	292	199	292	173	57	83	198	285	205
**DRUDE**	294	168	64	81	208	288	201	290	179	58	134	189	259	234
**AMOEBA**	286	181	59	85	207	289	193	285	189	55	136	196	275	236

[a]Medians rather than averages were used due to the non-gaussian distribution of some of the dihedrals (see also Figure S3).

Supporting Information Figures

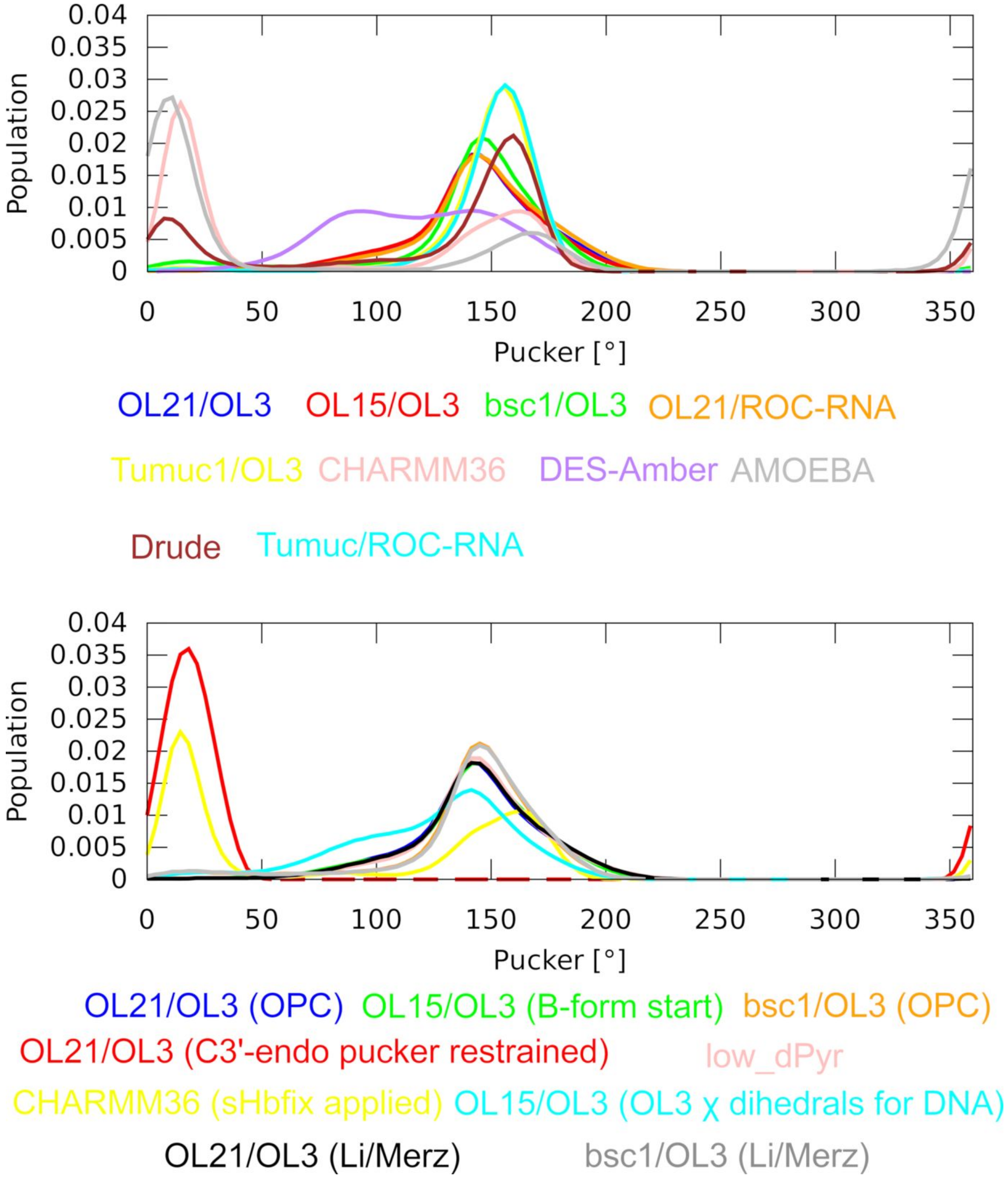

Figure S1. **Histograms of the DNA sugar puckers in MD simulations of the DD_hybrid structure and the low_dPyr structure.** Due to the sheer number of simulations, the datasets were separated into two graphs for the sake of clarity. The individual force-field combinations are color-coded for each graph according to the legend below the graph. For additional explanation of the data sets in the bottom graph, see the main text Methods and Table 1.

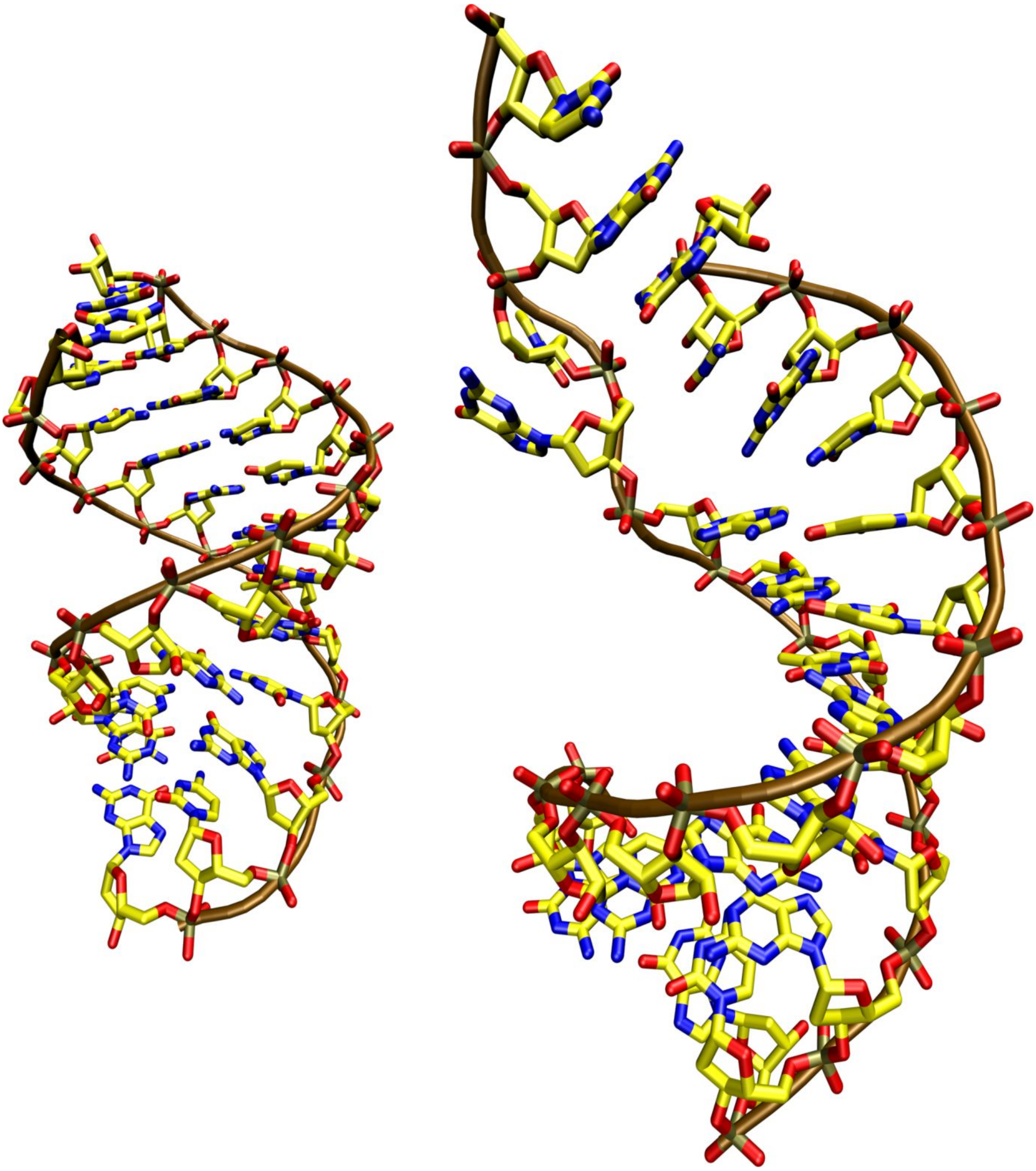

Figure S2. **Example of the extensive loss of base pairing sometimes observed in MD simulations using the CHARMM36 *ff*.** Two snapshots of the DD_hybrid structure are shown, a stable helix (left) and one with four consecutive GC base pairs disrupted in the upper part (right). The disruptions can be prevented by applying sHBfix on every base pair (see the main text and Table S3).

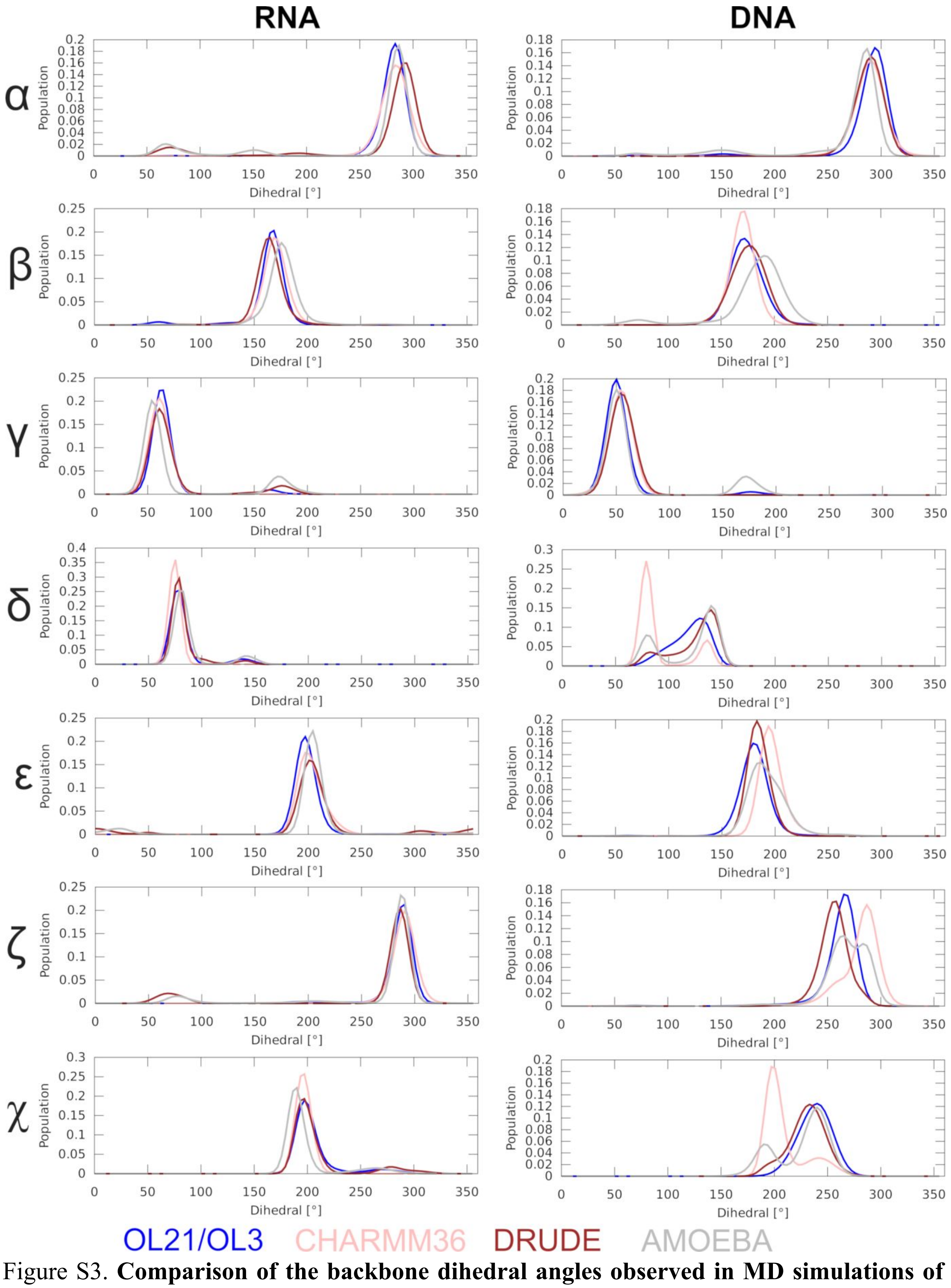

Figure S3. **Comparison of the backbone dihedral angles observed in MD simulations of the PPT hybrid structure using the polarizable and selected non-polarizable force fields.**

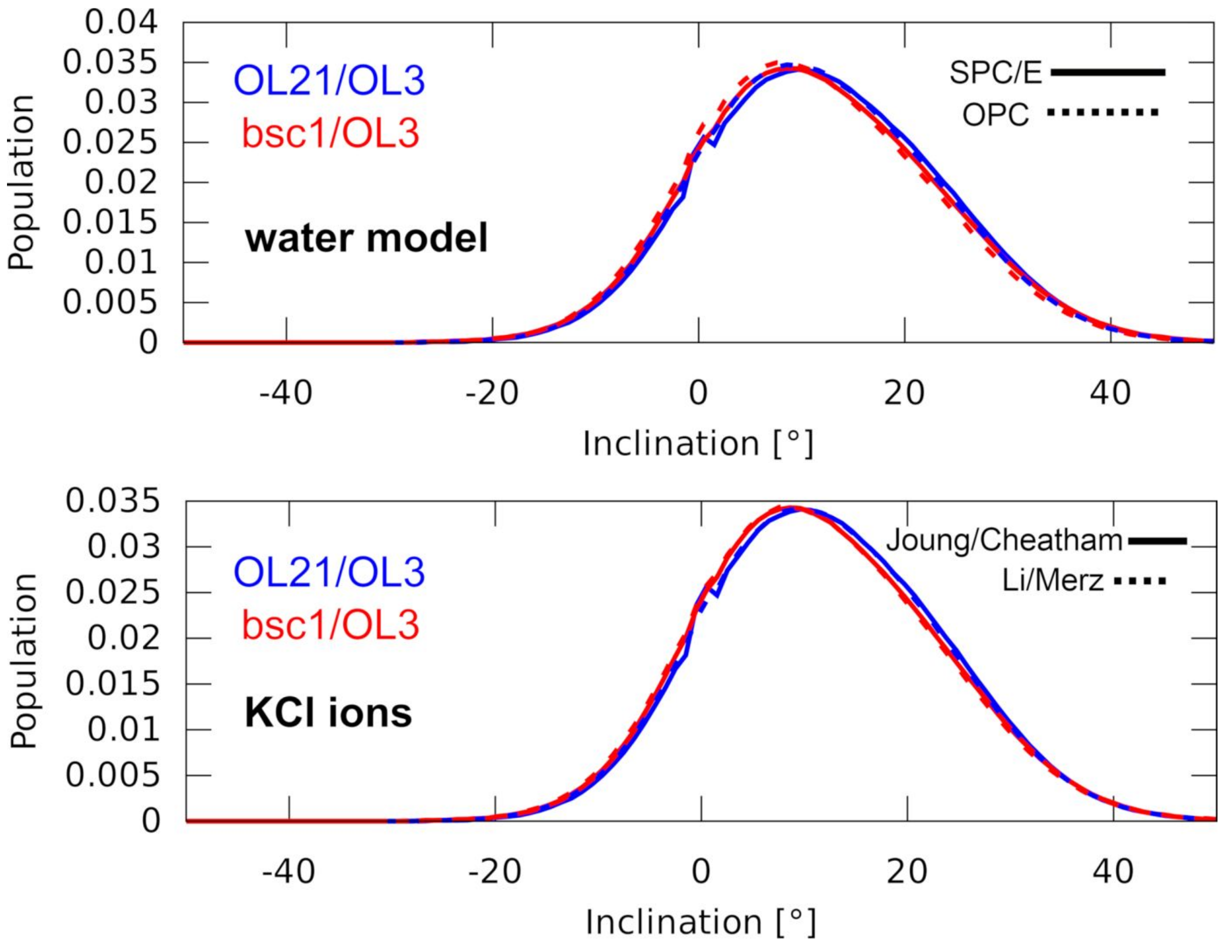

Figure S4. **Inclination of the DD_hybrid structure when simulated using the SPC/E and OPC water models (top) or the Joung/Cheatham and Li/Merz KCl ion parameters (bottom).** The individual force field and parameter combinations are color-coded and differentiated by the use of full or dashed lines, according to the legend.

Supporting Information References

(1) Li, P.; Song, L. F.; Merz, K. M., Jr., Systematic Parameterization of Monovalent Ions Employing the Nonbonded Model. *J. Chem. Theory Comput.* **2015**, *11*, 1645-1657.

(2) Jurečka, P.; Zgarbová, M.; Černý, F.; Salomon, J., Multistate B- to A- Transition in Protein-DNA Binding – How Well is it Described by Current AMBER Force Fields? *J. Biomol. Struct. Dyn.* **2024**, 1-11.

(3) Kopka, M. L.; Lavelle, L.; Han, G. W.; Ng, H.-L.; Dickerson, R. E., An Unusual Sugar Conformation in the Structure of an RNA/DNA Decamer of the Polypurine Tract May Affect Recognition by RNase H. *J. Mol. Biol.* **2003**, *334*, 653-665.

(4) Kuhrova, P.; Best, R.; Bottaro, S.; Bussi, G.; Sponer, J.; Otyepka, M.; Banas, P., Computer Folding of RNA Tetraloops: Identification of Key Force Field Deficiencies. *J. Chem. Theory Comput.* **2016**, *12*, 4534–4548.
